# *AgeWell.de* – study protocol of a pragmatic multi-center cluster-randomized controlled prevention trial against cognitive decline in older primary care patients

**DOI:** 10.1186/s12877-019-1212-1

**Published:** 2019-08-01

**Authors:** Andrea Zülke, Tobias Luck, Alexander Pabst, Wolfgang Hoffmann, Jochen René Thyrian, Jochen Gensichen, Hanna Kaduszkiewicz, Hans-Helmut König, Walter E. Haefeli, David Czock, Birgitt Wiese, Thomas Frese, Susanne Röhr, Steffi G. Riedel-Heller

**Affiliations:** 1 Institute of Social Medicine, Occupational Health and Public Health (ISAP), Medical Faculty, University of Leipzig, Philipp-Rosenthal-Strasse 55, 04103 Leipzig, Germany; 2 Department of Economic & Social Sciences & Institute of Social Medicine, Rehabilitation Sciences and Healthcare Research (ISRV), University of Applied Sciences Nordhausen, Nordhausen, Germany; 3 Institute for Community Medicine, University Medicine Greifswald (UMG), Greifswald, Germany; 4 German Center for Neurodegenerative Diseases (DZNE), site Rostock/ Greifswald, Greifswald, Germany; 5 Institute of General Practice/Family Medicine, University Hospital of LMU Munich, Munich, Germany; 6 Institute of General Practice, University of Kiel, Kiel, Germany; 7 Department of Health Economics and Health Service Research, University Medical Center Hamburg-Eppendorf, Hamburg, Germany; 8 Department of Clinical Pharmacology and Pharmacoepidemiology, University Hospital Heidelberg, Heidelberg, Germany; 9 Institute for General Practice, Work Group Medical Statistics and IT-Infrastructure, Hannover Medical School, Hannover, Germany; 10 Institute of General Practice and Family Medicine, Martin-Luther-University Halle-Wittenberg, Halle, Saale Germany

**Keywords:** Prevention, Multi-component intervention, Lifestyle, Cognition, Mental health, Dementia, Primary care, Cluster-randomized controlled trial, Late life

## Abstract

**Background:**

In the absence of treatment options, the WHO emphasizes the identification of effective prevention strategies as a key element to counteract the dementia epidemic. Regarding the complex nature of dementia, trials simultaneously targeting multiple risk factors should be particularly effective for prevention. So far, however, only few such multi-component trials have been launched, but yielding promising results. In Germany, comparable initiatives are lacking, and translation of these complex interventions into routine care was not yet done. Therefore, *AgeWell.de* will be conducted as the first multi-component prevention trial in Germany which is closely linked to the primary care setting.

**Methods:**

*AgeWell.de* will be designed as a multi-centric, cluster-randomized controlled multi-component prevention trial. Participants will be older community-dwelling general practitioner (GP) patients (60–77 years; *n* = 1,152) with increased dementia risk according to CAIDE (Cardiovascular Risk Factors, Aging, and Incidence of Dementia) Dementia Risk Score. Recruitment will take place at 5 study sites across Germany. GP practices will be randomized to either intervention A (advanced) or B (basic). GPs will be blinded to their respective group assignment, as will be the statistician conducting the randomization. The multi-component intervention (A) includes nutritional counseling, physical activity, cognitive training, optimization of medication, management of vascular risk factors, social activity, and, if necessary, further specific interventions targeting grief and depression. Intervention B includes general health advice on the intervention components and GP treatment as usual. We hypothesize that over the 2-year follow-up period the intervention group A will benefit significantly from the intervention program in terms of preserved cognitive function/delayed cognitive decline (primary outcome), and other relevant (secondary) outcomes (e.g. quality of life, social activities, depressive symptomatology, cost-effectiveness).

**Discussion:**

*AgeWell.de* will be the first multi-component trial targeting risk of cognitive decline in older adults in Germany. Compared to previous trials, *AgeWell.de* covers an even broader set of interventions suggested to be beneficial for the intended outcomes. The findings will add substantial knowledge on modifiable lifestyle factors to prevent or delay cognitive decline.

**Trial registration:**

German Clinical Trials Register (reference number: DRKS00013555).

## Background

### Relevance

Dementia is not only burdensome for the individuals affected and their relatives, it is also a major public health concern [1]. A substantial increase in the absolute number of people affected is to be expected due to dramatic demographic changes [1, 2]. So far, most types of dementia, particularly Alzheimer’s dementia (AD) as the most common type (60–70% of cases; [3]), cannot be cured. Therefore, the identification of effective strategies for prevention has been emphasized as a key element to counteract the dementia epidemic [4, 5].

Numerous vascular and lifestyle factors have been linked to dementia and AD [6, 7], suggesting that 35% of current dementia cases could be attributed to nine modifiable risk factors (midlife hypertension and obesity, diabetes mellitus, depression, physical inactivity, smoking, low educational attainment, hearing loss, and social isolation [8–11]. Similar results were found for Germany, suggesting a tremendous potential for prevention [10]. Indeed, recent studies indicated trends towards lower dementia incidence in Western high-income countries, likely being the result of improvements in such modifiable lifestyle factors [12]. Further modifiable risk factors may include the use of anticholinergic drugs [13] and atrial fibrillation [14]. Previous randomized controlled trials (RCTs) on interventions aiming at preventing cognitive decline, however, mainly focused on the management of single such risk factors [7]. Regarding the complex nature of dementia as well as the concurrence and interaction of the underlying risk factors, RCTs simultaneously targeting multiple risk factors should be more effective [7]. Large international multi-component trials [15–17] yielded very promising results in this regard.

The *Finnish Geriatric Intervention Study to Prevent Cognitive Impairment and Disability* (FINGER) was the first large RCT providing evidence that a multi-component intervention can be effective in improving or maintaining cognitive functioning in older individuals [5, 16]. Individuals aged 60–77 years (*n* = 1,200) with an increased dementia risk were randomly assigned to receive either a 2-year multi-component intervention (nutritional counseling, physical activity enhancement, cognitive training, vascular risk monitoring) or regular health advice (control group). The primary outcome was change in cognition as measured through a comprehensive neuropsychological test battery (NTB) [16]. During the 2-year follow-up period, cognition improved significantly in the intervention group compared to the control group [5].

In Germany, comparable initiatives have been lacking so far. Thus, the *AgeWell.de* study is designed to first-time investigate prevention potential for cognitive decline through a multi-component intervention, with a focus on modifiable risk factors such as, for example, lifestyle interventions, in older primary care patients in Germany.

### Objectives

**Fig. 1:**
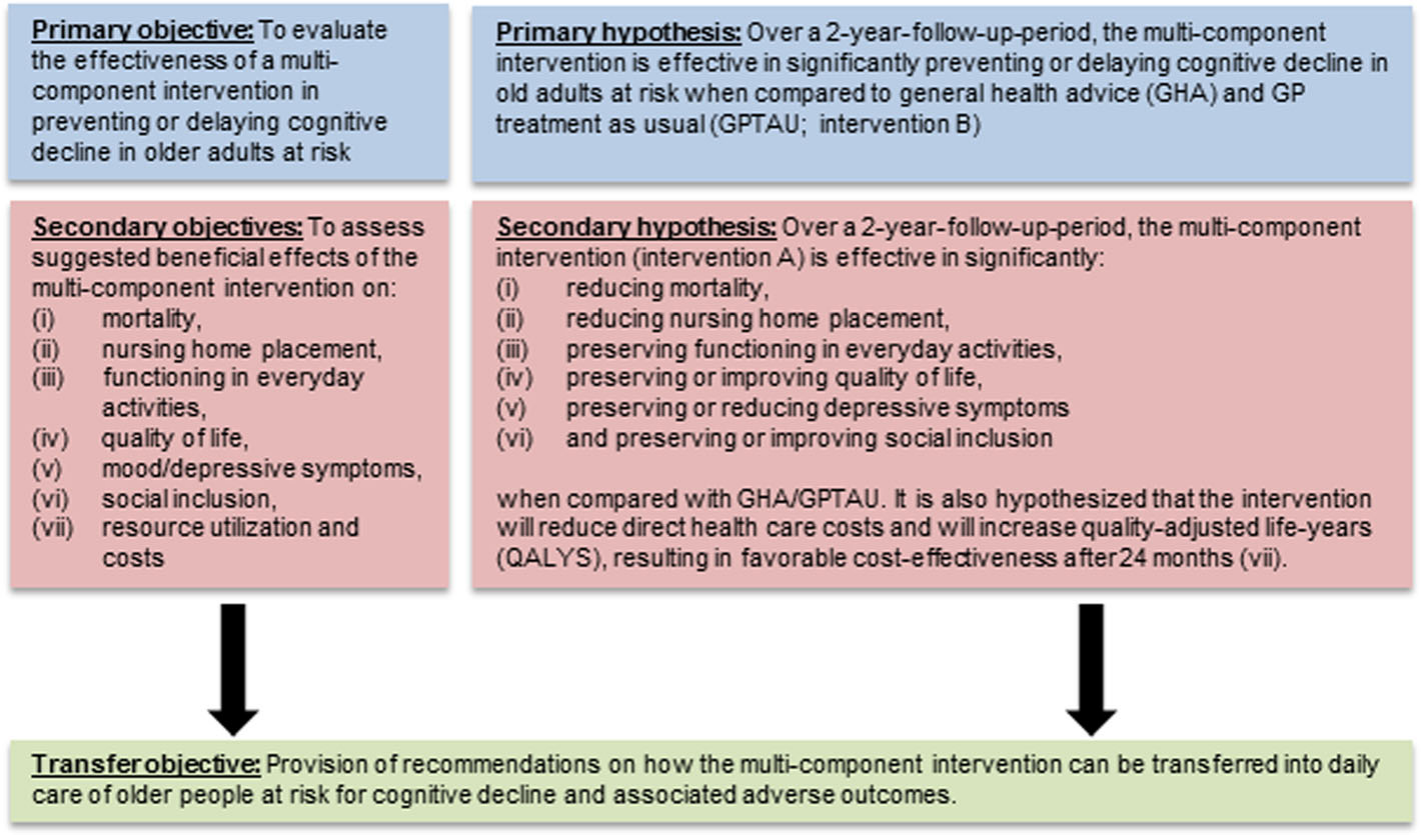
Study aims and hypotheses of AgeWell.de

The *primary objective* of *AgeWell.de* is to evaluate the effectiveness of a multi-component intervention in preventing or delaying cognitive decline in older adults at increased risk for dementia, specifically tailored to the German health care context. To do so, a pragmatic multi-centric, cluster-randomized controlled prevention trial will be conducted in primary care. The *secondary objective* is to assess effects of the multi-component intervention regarding (i) mortality, (ii) nursing home placement, (iii) functioning in everyday activities, (iv) quality of life, (v) depressive symptoms, (vi) social inclusion, and (vii) the cost-effectiveness of the intervention. A detailed overview of the study aims and associated hypotheses is provided in Fig. 1.

On the one hand, our study will be guided by the FINGER trial by including the components nutritional counseling, physical activity enhancement, cognitive training, and vascular risk monitoring in the 2-year intervention program. On the other hand, we aim to additionally include components of social lifestyle and recommendations on medication overuse and underuse. Encouraging participants to be socially active should also be a beneficial component of the intervention as even in old age an active lifestyle is protective against dementia [6, 18–20]. Likewise, we will provide specific recommendations on the medications of the study participants to their respective GP, if needed. German health claims data suggested that 22–25% of individuals aged 64 years and older receive at least one prescription of a potentially inappropriate medication (PIM) drug within one year [21, 22]. Primary data from a sample of community-dwelling people with dementia indicate that 22% receive at least one PIM with the highest prevalence being antidepressants, benzodiazepines and analgetics [23]. PIM constitutes a major public health concern especially in older age leading, for example, to an increased risk of adverse drug reactions, hospitalization, and mortality [24, 25]. Moreover, PIM is of particular importance as studies strongly suggest that specific drugs (e.g. with anticholinergic properties) can also increase the risk for dementia [13, 26, 27]. On the other hand, medication underuse can modify cardiovascular and other risks [28] and thus outcomes relevant for dementia development.

Finally, we will address further known risk factors for dementia by providing specific interventions in case of bereavement, grief, and depressive symptoms [29, 30], if necessary. The broad range of outcomes will additionally enable the estimation of total benefit and cost-effectiveness of the intervention suggested to be more beneficial for the intended outcomes. The study protocol describes the rationale and the study design of the AgeWell.de trial in adherence to the SPIRIT 2013 statement (Standard Protocol Items: Recommendations for Interventional Trials; [31]).

## Methods

### Study design

**Fig. 2:**
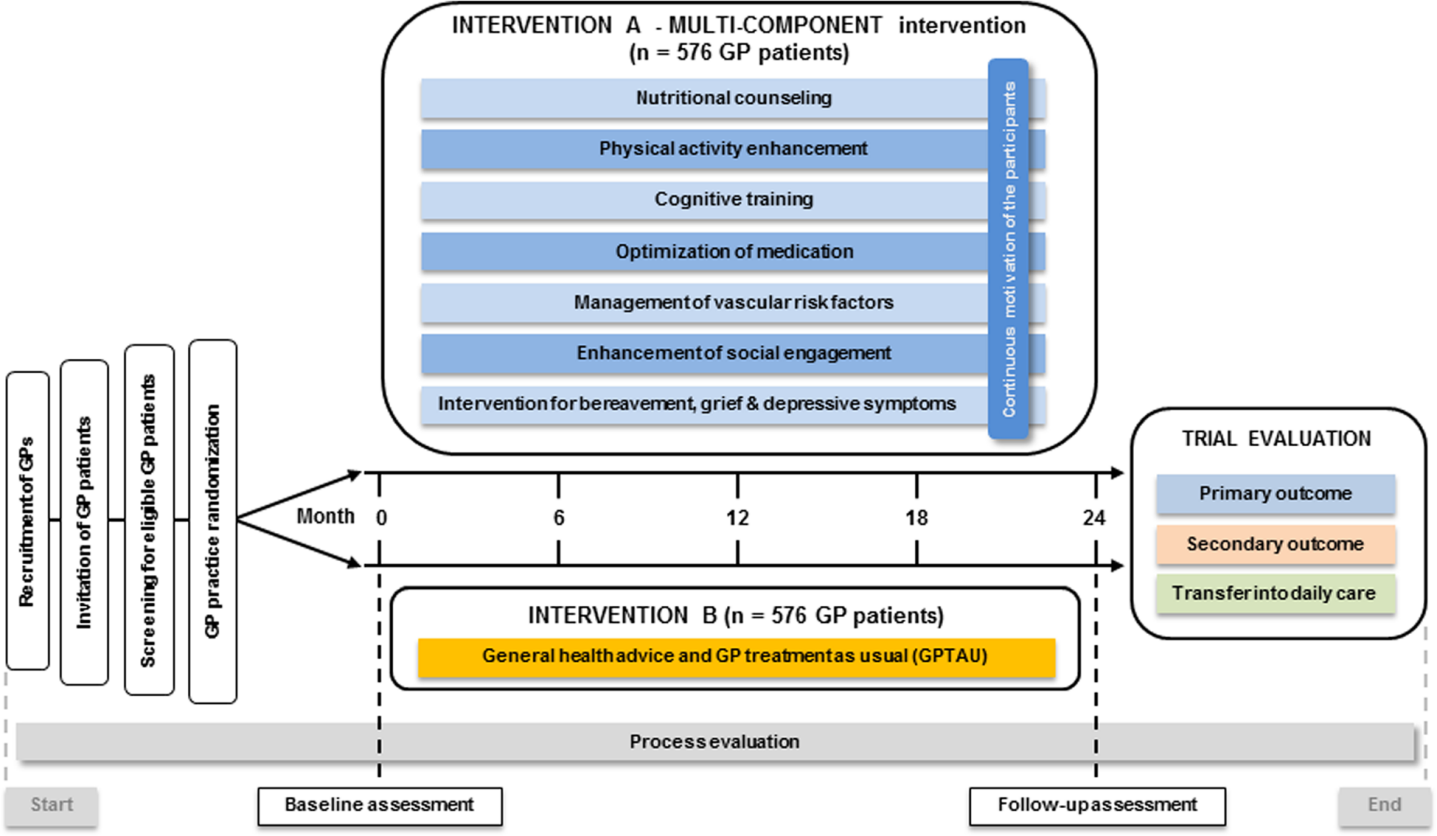
AgeWell.de study design

To evaluate the effectiveness of a multi-component intervention in preventing or delaying cognitive decline in older adults at risk, a multi-centric cluster-randomized controlled trial with primary care patients will be conducted (Fig. 2).

### Inclusion and exclusion criteria

To target individuals at risk for dementia and suitable for the preventive intervention program, we will include community-dwelling GP patients who are 60–77 years old and have an increased CAIDE Dementia Risk Score (Cardiovascular Risk Factors, Aging, and Incidence of Dementia; [16, 32]). We will use a CAIDE Dementia Risk Score ≥ 9 for inclusion. The risk score predicted dementia risk with a sensitivity of 0.77 and a specificity of 0.63, when a cut-off ≥9 was applied [32]. CAIDE is based on information that is easy to assess (age, education, gender, blood pressure, body mass index, total cholesterol, and physical activity). Therefore, case finding of eligible participants for *AgeWell.de* can be easily conducted in the GP practices. Moreover, the procedure could be transferred into daily care later on.

Exclusion criteria are conditions affecting safe engagement in the intervention (malignant disease/fatal illness, severe clinical depression, symptomatic cardiovascular disease, revascularization within the previous year) as judged by the GP; severe loss of vision, hearing, or communicative ability/insufficient ability to speak and read German; severe mobility impairment; concurrent participation in another intervention trial, and previously diagnosed dementia or dementia suspected by the GP.

### Interventions

During the 2-year follow-up period, participants of intervention group A (advanced) will receive a systematic comprehensive multi-component intervention program (Table 1). All intervention components will be delivered face-to-face by the study nurses during a visit at the participants’ homes. The intervention will include

- advice on healthy nutrition, based on the guidelines of the German Nutrition Society (DGE)
- exercises for strength, balance and flexibility on two days per week; aerobic training (3–5 days per week for 20–30 min) planned individually with the participant
- cognitive training with tablet computers, using the cognitive training software “NeuroNation”, three times per week
- enhancement of social engagement, planned individually with the participant
- if necessary, feedback on vascular risk factors (e.g. smoking, medical history) and ways to reduce the respective risk
- assessment of depressive symptoms and underlying risk factors (e.g. bereavement, grief); if necessary, patients will be encouraged to contact their GP who will provide adequate support and care (e.g. referring participants to groups, psychiatrists, psychotherapists, psychiatric hospitals); written information on addresses and helplines which can be contacted in case of grief and/or depressive symptoms.
- optimization of medication: see Table 1 for a detailed description.

**Table 1:** Components of the *AgeWell.de* intervention program (intervention group A)

Intervention Component	Description
Nutritional counseling	- Based on the guidelines of the *German Nutrition Society* [33] including recommendations e.g. on intake of cereal products, fruit and vegetables, fish, sugar, salt, and of fluid. - Anthropometric measurements (height, weight, BMI)
Physical activity enhancement	- Combined training program including (i) muscle-strengthening activity, (ii) flexibility activity/balance exercise, on 2 days/week, and (iii) aerobic activity (3–5 days a week for 20–30 min). Strength and flexibility/balance training can be conducted at home. Aerobic training will be planned with each participant individually - Participants will receive a pedometer to record the number of steps walked daily
Cognitive training	- Information on cognitive functioning and the impact of cognitive activity/training on cognitive performance and dementia risk; information on strategies to train cognition in everyday life - Cognitive training at home on a regular basis (3 times/week, 15 min per session) with tablet computers (software: NeuroNation; https://www.neuronation.com/)
Optimization of medication	- Collection of baseline information (i) from the GP on participants’ medication, diagnoses, and lab values (creatinine, hemoglobin A1c) and (ii) from the participants on their actual medication - Electronically supported data evaluation to identify potentially inappropriate medication (e.g. anticholinergics, using a list based on Gray and coworkers [13], potentially missing drugs (using START criteria A1 to A8; [34]), contraindicated drug combinations, and contraindications in patients with renal impairment (using databases from the electronic drug information system AiD*Klinik*). Discrepancies in the information on medication collected from the participant and the GP will be identified. - Reports including recommendations on the medication of a participant will be transmitted to the GP. For recommended drugs, this will include information on potentially serious drug-drug interactions and on drug dose adjustment in patients with renal impairment. A procedure is established in case of emergencies, e.g. if an important drug for a serious condition is not administered by the patient. If a patient reports difficulties with drug administration (difficulties swallowing tablets or capsules, tablet splitting, subcutaneous injections, use of inhalers, transdermal patch application, or with the administration of eye drops, nose drops, ear drops, or rectal or vaginal drug administration), specific information is provided to the patient.
Management of vascular risk factors	- Assessment of medication and diagnoses, lab values, health parameters, lifestyle factors, blood pressure and anthropometric measurements (height, weight) - Information on further vascular risk factors (e.g. smoking, medical history) - Feedback on vascular risk, importance of reducing the risk, and possible ways of achieving such a reduction - Nutritional counseling, recommendation of weight loss (if necessary), physical activity enhancement, and optimization of medication as described above
Enhancement of social engagement	- Assessment of level of social activity and risk of social isolation - Information on the importance of an active lifestyle including high social engagement for dementia risk - Enhancement of social engagement will be planned together with each study participant individually
Bereavement, grief and depressive symptoms	- Assessment of depressive symptoms and underlying risk factors (e.g. bereavement, grief) - If necessary, participants will be encouraged to contact their GP and will be provided with adequate support and care (e.g. referring participants to groups, psychiatrists, psychotherapists, psychiatric hospitals). Additionally, participants will receive written information on addresses and helplines which can be contacted in case of grief and/or depressive symptoms. - If applicable, encouragement to use MoodGym – a scientifically developed and evaluated free web-based training program to prevent and reduce depressive symptoms (http://www.moodgym-deutschland.de/).
Motivational tasks across all components	- Personnel continuity regarding the contact person for the participants - Study nurses will be trained in motivational interviewing techniques and participants will be strongly encouraged to contact their contact person, whenever necessary - Birthday/Christmas/holiday cards originally signed by trial authorities/principal investigators - Participants of intervention A will receive a brochure including recommendations (additional tips and suggestions each week for an active lifestyle, e.g. recipes, suggestions for physical activity, and information on healthy ageing) as well as a weekly diary to track their activities in the intervention components.

Participants of intervention group B (basic) will receive *GP treatment as usual* (GPTAU) and *general health advice* (GHA) related to the components of intervention A. We hypothesize that the multi-component intervention program of intervention A will be superior to GPTAU and GHA (intervention B) regarding trial outcomes.

### Outcomes

#### Primary endpoint

In accordance with previous trials [2, 5, 7], we will assess change in cognitive performance as the primary endpoint. Cognitive performance will be assessed with a neuropsychological test battery covering the six cognitive domains for diagnosing mild and/or major neurocognitive disorder according to DSM-5 (attention, executive function, learning/memory, language, perceptual-motor abilities, and social cognition). Composite cognitive *z-*scores based on the results from all single tests will be calculated. Single *z-*scores will be calculated using baseline mean and standard deviations and then, for the composite score, the single *z-*scores will be averaged.

#### Secondary endpoints

An overview of secondary endpoints (mortality, nursing home placement, instrumental activities of daily living/activities of daily living, quality of life, depressive symptoms, social inclusion, motivation for behavior change, cost-effectiveness) is provided in Fig. 1. Corresponding assessments are detailed in Table 2. Furthermore, we will assess readiness for behavior change and explore mediating and moderating factors for the effectiveness of the intervention.

**Table 2:** Instruments used in *AgeWell.de*

*Measures of cognitive performance (primary endpoint)*	
Construct	Instrument
*Cognitive performance* ^a^	• Trail Making Test A and B [35] • Word List Memory - CERAD subtest [36–38] • Verbal Fluency Test - Animals - CERAD subtest [37–40] • Constructional Practice – CERAD subtest [37, 38, 41] • Reading the Mind in the Eyes Test - Revised version [42, 43]^b^ • Montreal Cognitive Assessment (MoCA; [44]).
*Instruments to assess secondary endpoints in AgeWell.de*	
Construct	Instrument
*Mortality*	Information obtained from the GP or confidant elected by the participant [self-constructed items^c^]
*Nursing home placement*	Information from the participant or – if the participant is unavailable or dead – from the GP or a contact person elected by the participant
*ADL/IADL*	Barthel Index [45], Amsterdam-IADL scale [46]^a^
*Quality of life*	EQ-5D and visual analogue scale (EQ VAS scale) [47]; WHOQOL-Bref [48], WHOQOL-Old [49]
*Depressive symptoms* ^a^	Geriatric Depression Scale (GDS; [50, 51])
*Social inclusion*	Lubben Social Network Scale (LSNS; [52] in combination with standardized questionnaire on social activity [self-constructed items^c^]
*Cost effectiveness*	Fragebogen zur Inanspruchnahme medizinischer und nicht-medizinischer Versorgungsleistungen im Alter (FIMA)-Questionnaire for Health-Related Resource Use in an Elderly Population [53]; unit costs for monetary valuation of resource use [54]; EQ-5D [47]
*Motivation for behavior change*	Stage assessment in the adoption and maintenance of physical activity and fruit and vegetable consumption [55–57]
*Instruments to assess further information relevant for the AgeWell.de-trial*	
Construct	Instrument
*Sociodemographic information*	Standardized questionnaire (age, sex, educational level/professional life/activity, living situation/marital status, socio-economic status)
*Subjective cognitive decline* ^a^	Standardized questionnaire on subjective cognitive decline [58]
*Self-reported impairments and symptoms*	Standardized questionnaire on self-reported impairment in walking, vision, or hearing [self-constructed items^c^] Standardized questionnaire on self-reported anticholinergic symptoms [self-constructed items^c^]
*Anthropometry, blood pressure*	Measurement of height, weight, blood pressure; calculation of body-mass-index (BMI)
*Nutrition*	Standardized questionnaire on food consumption (food frequency questionnaire/FFQ [59])
*Physical activity I*	Standardized questionnaire on physical activity [self-constructed items^c^]
*Bereavement, grief*	Standardized questionnaire on bereavement [60]
*Medication I*	(i) Information from the attending GP on participants’ medication (“GP-list”) and diagnoses^b^, lab values using GP practice records and (ii) information from participants on their actual medication (“brown-bag review”), adherence, and difficulties with drug administration using standardized questionnaires [self-constructed items^c^]
*additional vascular risks*	Standardized questionnaires on additional vascular risk factors (e.g. smoking, medical history, familial medical history; [self-constructed items^c^])
*Instruments to assess further information in intervention group A*	
Construct	Instrument
*Physical activity II*	Weekly records on conduct of the physical activity intervention component, training, and pedometer results [self-constructed items^c^]
*Cognitive training*	Weekly records on conduct of the cognitive training [self-constructed items^c^]
*Medication II*	Standardized feedback questionnaires on potential changes in a participant’s medication or reasons for not following the recommendations, completed by the attending GP [self-constructed items^c^]
*Social activity*	Weekly records on social activity, in addition to the information collected on social inclusion (see above)
*Motivation and readiness for change*	Standardized questionnaire on motivation and readiness for behavior change [55–57]
*Instruments to conduct the process evaluation*	
Construct	Instrument
*Success rate of recruitment and quality of the study population*	Standardized questionnaires filled out by the GP practice personnel (number of eligible GP patients, number of participants and non-participants, (baseline) characteristics of participants and non-participants, reasons for non-participation) [self-constructed items^c^]
	Standardized telephone interview on reasons for leaving the study with intervention A-participants, if applicable [self-constructed items^c^]
*Quality of the execution of the intervention, burden for GP patients and GPs*	Standardized interviews with all intervention A-participants on adherence to the intervention components and potential burdens [self-constructed items^c^]
	Standardized GP questionnaires on barriers and facilitators for adherence to intervention components in the GPs’ view and on own potential burdens [self-constructed items^c^]

*GP* general practitioner, *ADL* Activities of Daily Living, *IADL* Instrumental Activities of Daily Living

^a^Information should be also used to diagnose DSM-5 Mild and Major Neurocognitive Disorder/dementia

^b^The composite cognitive z-score for the primary endpoint change in cognitive performance will be calculated based on the test results regarding these six domains

^c^Questionnaires can be obtained from the corresponding author upon request

### Sample size

Sample size calculations are based on a composite *z-*score of cognitive performance from previous findings in mild AD [61], i.e., a mean decrease in the composite *z-*score of cognitive performance of − 0.21 with a common SD of 0.5 is assumed in intervention group B during the two year follow-up period. Accordingly, the required sample size is estimated to be 475 participants per group in order to detect a 50% difference in change in the composite *z*-score between intervention groups (2-sided *t*-test for equal variances; with 5% significance level and 90% power). We assume an inflation factor of 1.1 (corresponding to an intra-cluster correlation coefficient of 0.02 and a cluster size of 6). In view of the findings from the FINGER trial, we further assume a dropout rate of no higher than 10% [5]. Therefore, a total sample size of *n* = 1,152 individuals (*n* = 576 per group) seems sufficient.

### Recruitment procedure

**Fig. 3:**
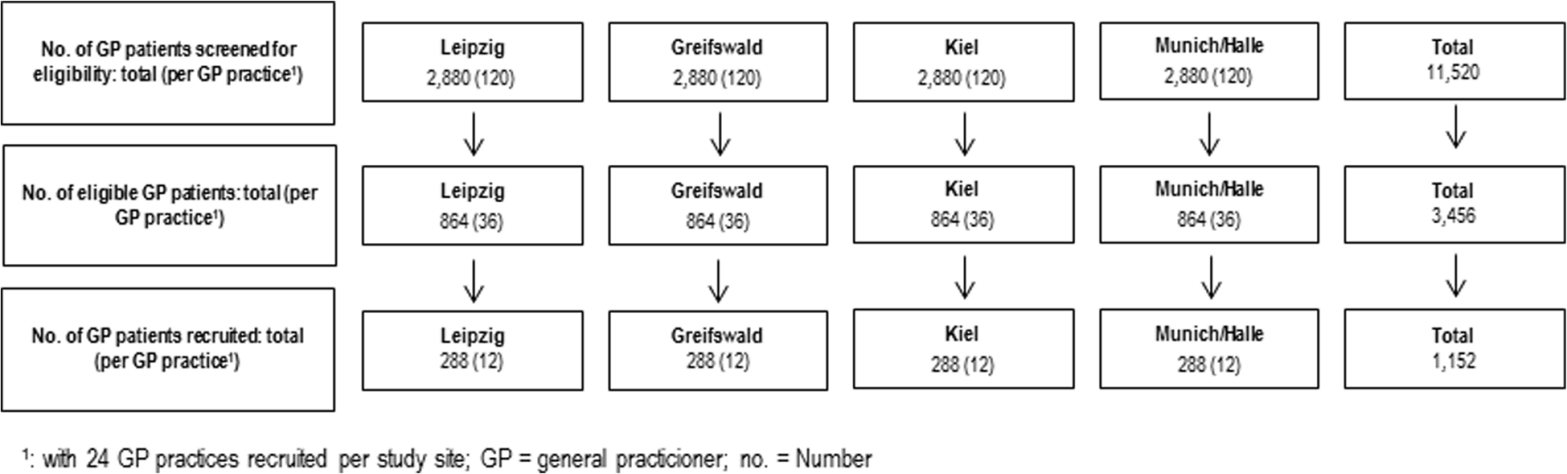
AgeWell.de recruitment of participants per study site

To ensure the inclusion of 1,152 patients, recruitment will take place at five study sites across Germany (Greifswald, Kiel, Leipzig, and Munich/Halle; Fig. 3). We estimate that (i) 30% of the GP patients between 60 and 77 years are eligible for the study (according to the FINGER trial, approximately 40% have the required CAIDE Dementia Risk Score ≥ 9 [30]; among these another 25% are estimated to be excluded according to the *AgeWell.de* exclusion criteria.). Based on previous experiences from other multi-centric trials [62, 63] and regarding the demanding nature of this prevention trial, we assume a rather conservative response rate of approximately 33%. Thus, every study site will screen *n* = 2,880 GP patients to identify *n* = 864 potentially eligible GP patients (except for Munich/Halle: half numbers due to shared recruitment between the two study sites). To ensure these numbers, we suggest a number of *n* = 24 GP practices per study site (*n* = 96 GP practices in total) as sufficient, leading to a total number of *n* = 12 patients that have to be recruited per GP practice. GP practices will receive monetary incentives for recruitment and provision of patients’ data.

### Randomization

To ensure a balance in sample size across groups over time, block-randomization of the GP practices will be used with a targeted assignment ratio of participants to intervention A vs. B of 1:1. Participating GP practices (clusters) will be randomized either to intervention A or B. Randomization will be conducted at the data management center at the Institute for General Practice at the Hannover Medical School, using a randomization list that is concealed to the recruiting study sites.

### Blinding

Whereas blinding of interviewers and study nurses cannot be realized in *AgeWell.de*, GPs will be blinded towards their respective group allocation. Eligible participants will receive all necessary information about the study in an information sheet provided by the GP, including information on the components of both interventions and on the random assignment of participants to either intervention A or B. After signing an informed consent at the GP’s practice, participants will receive a letter from the study sites with information on their respective group allocation and on next steps of the study.

### Data collection

Each study center will be recruiting GP practices using an invitation letter with information on study design and aims as well as GPs’ duties during the trial. GPs interested in study participation can reply per fax or telephone. The recruiting study sites will then schedule a personal appointment at the GP practice to explain the recruitment procedure and to provide all necessary documents (informed consent form, patient information, screening sheets etc.).

Case finding and recruitment will be conducted by the GP practices. Data of regular GP patients will be screened by trained practice personnel in association with the *AgeWell.de*-study personnel, according to the inclusion and exclusion criteria. The GP will then provide all necessary information about the study to eligible patients. Patients who are interested in study participation will sign an informed consent. Contact information of the patients will be sent to the local recruiting centers. Next, patients who have consented to participate will receive a letter from the study personnel, informing them about their respective group allocation. Home visits for baseline assessment will be scheduled.

At baseline and at follow-up assessment after 24 months, fully structured interviews will be conducted with all participants, assessing the primary and secondary endpoints. Other relevant information assessed include sociodemographic information, subjective cognitive decline, self-reported impairments and symptoms, anthropometry, blood pressure, nutrition, physical activity, bereavement/grief, medication, vascular risks). Moreover, GPs will provide data on participants’ health characteristics (diagnoses, lab values, medication) using standardized questionnaires. To conduct the process evaluation, the success rate of recruitment (number of eligible GP patients, number of participants and non-participants, (baseline) characteristics of participants and non-participants, reasons for non-participation) will be provided by the GP practice personnel. Standardized telephone interviews on reasons for dropout will be held with intervention A-participants, if applicable. For participants in intervention A, additional interim sessions will be scheduled: one face-to-face assessment after 12 months at the participants’ home and five telephone calls after 2, 4, 8, 16, and 20 months, respectively (see Table 3). These fully structured interviews serve the purpose of monitoring adherence to the intervention and motivation (monitoring & booster sessions). Participants in intervention group A will be asked to track their activities in the intervention components using weekly diaries. To reduce non-response as well as dropout in *AgeWell.de*, interviewers will undergo training regarding all procedures and assessments, including motivational interviewing techniques [64]. Likewise, motivational tasks will also be carried out by the principal investigators (Table 1).

**Table 3:**
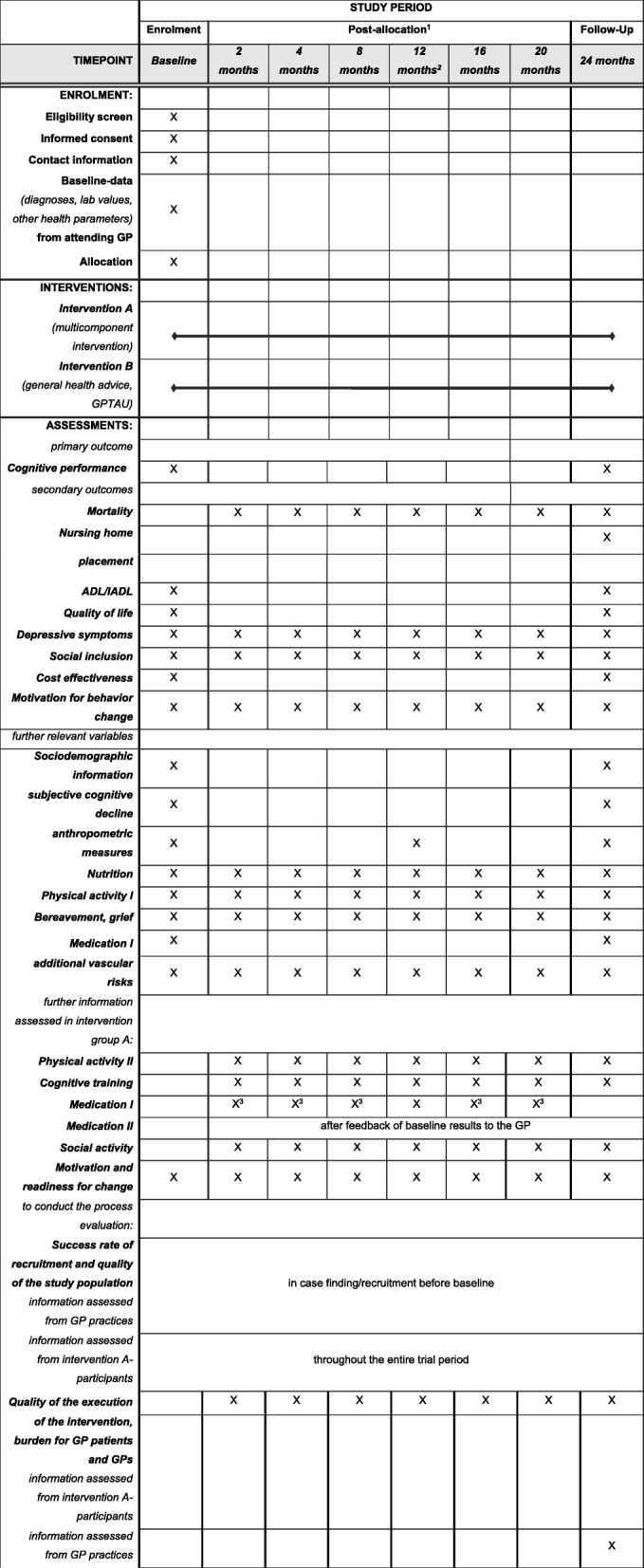
Schedule of enrolment, interventions and assessments in the course of *AgeWell.de*

^1^: Telephone interim monitoring and booster sessions 2, 4, 8, 16, and 20 months after baseline-assessment in intervention group A; ^2^: Face-to-face intervention visit in intervention group A after 12 months; ^3^: limited assessment of the actual medication via telephone

### Data management

Each local recruiting center will enter collected data via an internet-based electronic data capture system which complies with the FDA requirements (21 CFR Part 11) as well as the guidelines for Good Clinical Practice (GCP). Data will be stored in a central Oracle-database. For statistical analyses, data can be exported via a web-based export-tool into SPSS, SAS, CDISC ODM, or similar.

The technical infrastructure includes the internet-based remote data entry system and the central database server. Data will not be stored locally. For transfers, data will be secured using 128 bit SSL encryption. Access to the database and webserver is controlled by two consecutive firewall systems. Data will only be stored using a pseudonym generated automatically upon first entry of a patient’s data into the database. Access to the electronic data entry system will be provided to members of the *AgeWell.de* study group according to a detailed concept of roles and rights.

### Statistical analyses

Preliminary analyses involve three parts. First, data will be cleaned and checked for inconsistencies by statisticians at the central data management center to ensure data accuracy and coherence. Second, outcome variables will be examined to identify potential outliers and leverage points. Third, missing data will be inspected and handled according to patterns of data missingness, e.g., by multiple imputation [65].

A dropout analysis will be performed to test whether complete and incomplete cases differ according to relevant sociodemographic variables, potentially introducing selection bias.

Descriptive analyses will be carried out to examine differences in baseline measurements regarding the composite cognitive *z-*score (primary endpoint) and secondary endpoints between the two intervention groups. Additionally, single scores for the six neurocognitive domains will be calculated and analyzed. Suitable balancing techniques, such as entropy balancing, will be used for baseline measurements to warrant a high degree of comparability between study groups [66]. In order to analyze changes in primary and secondary endpoints of the two groups over time (treatment effect), latent growth curve modeling (LGM) will be utilized. Group membership (A vs. B) as well as other time-invariant characteristics will be assessed as predictors of change in cognitive performance in the models. Model estimates will be weighted using, for example, calculated entropy balancing weights to equalize possible baseline differences in covariates and outcomes. The models will further be stratified by age and gender to evaluate whether treatment effects on primary and secondary outcomes differ by subgroups.

Secondary analyses include, among others, investigation of effects of drug-associated risk factors. Effects of the intervention in patients with or without such factors will be analyzed by comparing primary and secondary endpoints in the respective subgroups. Plans for stratified analyses by overall cognitive performance as expressed by MoCA scores are effective. Cost-effectiveness will be analyzed based on both direct costs and quality-adjusted life-years (QALYs). To derive direct costs, health care utilization during 24 months follow-up will be valued using specific German unit costs. Intervention costs will be calculated using accounting principles. QALYs will be calculated using preference-based utilities as derived from the EQ-5D-5 L. Additionally, incremental cost-effectiveness ratios (ICER) will be calculated and net benefit regressions will be conducted to determine the uncertainty of the point estimates of the ICER [67].

### Monitoring

#### Data monitoring

A data monitoring committee will not be established as the overall risk associated with the trial is considered low, and therefore, the likelihood for the need to modify or discontinue the trial is considered insignificant. However, precautions have been taken to minimize the potential of any harms, as detailed below. Moreover, a detailed process evaluation will be conducted throughout the entire trial to produce in-depth insight into the delivery of the interventions, (i) to prevent drawing inappropriate conclusions on the efficacy or effectiveness of the interventions, and (ii) to formulate recommendations for future studies [68]. Most importantly, process evaluation should be used to identify and avoid potential burdens for study participants and GPs. The process evaluation will comprise three dimensions:

1. Evaluation of the success rate of recruitment: Standardized questionnaires filled in by the GP personnel collecting number of eligible patients, number of participants and non-participants, baseline-characteristics of both groups, and reasons for non-participation. Loss of participants during the follow-up-period as well as reasons for leaving the study will be assessed to evaluate attrition rate as well as barriers and facilitators for staying in the trial [5].
2. Evaluation of the quality of the execution of the intervention components: At every face-to-face or telephone interim session, adherence to the intervention will be assessed using standardized questionnaires with intervention A participants in order to identify potential barriers and facilitators for adherence to the intervention. Likewise, GPs will be asked to fill out a standardized questionnaire to identify potential burdens for participating GPs.
3. Evaluation of the process of data acquisition regarding completeness and validity: Documentation of reasons for discontinuing an assessment before completion as well as the motivation of the participants and the GPs for assessment. Number and characteristics of missing data will be analyzed. Information will be used to evaluate barriers and facilitators for data collection.

### Harms

Trial-related adverse events (AEs) will be assessed without regard towards a causal relation to the intervention. AEs will be recorded at each contact with participants after the baseline assessment. AEs in *AgeWell.de* include loss experiences and grief, depressive symptoms, severe injuries, hospitalization, and private or occupational stress reported by participants. Specific AEs (depressive symptoms; severe injuries; hospitalization) will be considered serious adverse events (SAEs) if they result in a life-threatening condition (immediate risk or death), hospitalization/prolongation thereof, or any lasting impairment. Occurrence of AEs/SAEs will be documented in the electronic database after each contact with participants. In case of a SAE, an automatic case-report form will notify the coordinating study center at the University of Leipzig, which, in turn, will contact the respective study center. A shared decision will then be made between the coordinating study center and non-elective principal investigator (PI) at the respective study center, including one of the following options: informing the participants’ GP about the SAE, interruption or discontinuation of trial participation, or none of the above.

### Auditing

Auditing will take place in form of reviews of the data collection across key assessment waves for each recruitment center. Specifically, 2 % of the questionnaires at baseline, at the face-to-face interim assessment (for intervention group A only), and at follow-up, respectively, will be randomly drawn and inspected regarding their degree of matching with the database input. Source data verification will be performed independently from investigators by the Hannover Medical School that is the entrusted site performing all data management tasks throughout the whole trial.

### Protocol amendments

Possible modifications to the protocol will be tracked in the German Clinical Trials Register (DRKS, registry number: DRKS00013555).

### Dissemination policy

*AgeWell.de* results will be published in scientific international, peer-reviewed journals, if possible with open access. Moreover, results will be presented at national and international scientific conferences as well as during seminars for GPs, regional care networks, and public events for seniors. Furthermore, publication results will be disseminated to the wider research community via press releases.

### Organizational structure and responsibilities

The Institute for Social Medicine, Occupational Health and Public Health (ISAP) in Leipzig acts as the coordinating center in *AgeWell.de*. This implies the organization of regular telephone-conferences and meetings with all participating centers, the compensation of all participating GP practices, preparation of documents needed for the conduct of the trial as well as reports to the funder of the study. Moreover, the study center in Leipzig also acts as one of the recruiting centers. As a subcontractor, the Institute for General Practice at the Hannover Medical School acts as the responsible study site for the task of data management, including the setup of the internet-based remote data capture system. The study centers in Greifswald, Kiel and Munich/Halle act as further recruiting study sites. Evaluation of cost-effectiveness of the trial will be conducted by the Department of Health Economics and Health Service Research at the University Medical Center Hamburg-Eppendorf. The intervention component “optimization of medication” will be realized by the Department of Clinical Pharmacology and Pharmacoepidemiology at the University Hospital Heidelberg, including the programming of algorithms to evaluate electronic data for optimization of medication and provision of documents for corresponding feedback to and from the GP.

## Discussion

Multi-domain interventions targeting lifestyle factors have been pointed out as a promising prevention strategy against dementia in international trials [5]. *AgeWell.de* will be the first such multi-component trial in older adults in Germany. We hypothesize that the multi-component intervention will be superior to general health advice and GPTAU in maintaining cognitive functioning in older adults at risk for dementia (primary hypothesis). Moreover, we assume beneficial effects in reducing mortality, nursing home placement, depressive symptoms, and in maintaining quality of life as well as functioning in everyday life (secondary hypotheses). Subsequently, this should also reduce direct health costs.

The *AgeWell.de*-study will add valuable insights regarding the role of modifiable risk and lifestyle factors to prevent or delay cognitive decline. A specifically useful asset should be findings from the intervention components that address potentially inappropriate medication [13, 26] and depression [8, 9, 69], which, to our knowledge, have not been examined in comparable RCTs so far.

Beyond that, our intervention will possibly also reduce risk for other diseases such as hypertension, stroke, cardiovascular disease, overweight etc. by addressing common risk factors for the respective diseases [2].

### Limitations

Participation in our prevention trial is rather demanding since the intervention components address various lifestyle factors and require e.g. cognitive training and physical activity on several days a week. The follow-up period is longer than in most previous trials testing single interventions. Therefore, adherence to the intervention might be lower compared to trials targeting only single components for a shorter time period. These factors also raise the chance of dropping out. We will address these potential problems by putting a strong focus on continuous motivation of participants and monitoring of adherence to the intervention, e.g. by regular telephone contacts and the use of motivational interviewing techniques. For ethical reasons, general health advice and feedback on known risk factors for dementia will also be provided to intervention group B. On these grounds, estimates of the effects of our intervention should be considered to be conservative.

### Outlook

Since the domains of our multi-component intervention address behaviors common in the general population, the intervention, if effective, will be implemented in real world settings. Thereby, *AgeWell.de* could add to targeted and cost-effective strategies in preventing dementia in older adults at risk. Most interventions tested (e.g. physical activity and a wholesome diet) are easily available to the majority of older adults [70]. Likewise, *AgeWell.de* addresses lifestyle factors that are common in general practice. Therefore, even a moderate decrease in exposure to the targeted lifestyle factors could lead to a significant reduction in incident dementia cases on a population level [5, 70]. Concerning the projected increase in number of dementia cases due to ageing populations, there is an evident need for effective prevention strategies. This becomes particularly relevant in regard to the absence of effective treatment options. To date, lifestyle interventions might constitute the most cost-effective and sustainable option for dementia prevention [71]. Against this background, the results of *AgeWell.de* will make a highly relevant contribution to the growing body of knowledge on modifiable risk factors for dementia.

## Data Availability

After publication of the final *AgeWell.de* trial results, electronic research data will be made accessible free of charge for third parties/further interested researchers upon request from the central data management center (work group Medical Statistics and IT-infrastructure at the Institute for General Practice, Hannover Medical School).

## Abbreviations

AD: Alzheimer’s disease
ADL: Activities of daily living
AE: Adverse event
BMBF: Bundesministerium für Bildung und Forschung (German Federal Ministry for Education and Research)
CAIDE score: “Cardiovascular Risk Factors, Aging, and Incidence of Dementia” Dementia Risk Score
CERAD: Consortium to Establish a Registry for Alzheimer’s Disease
DSM-5: Diagnostic and statistical manual of mental disorders, 5th edition
FINGER: Finnish Geriatric Intervention Study to Prevent Cognitive Impairment and Disability
GCP: Good clinical practice
GHA: General health advice
GP: General practitioner
GPTAU: General practitioner treatment as usual
IADL: Instrumental activities of daily living
ICER: Incremental cost-effectiveness ratios
LGM: Latent growth curve modeling
MoCA: Montreal Cognitive Assessment
NTB: Neuropsychological test battery
PI: Principal investigator
PIM: Potentially inappropriate medication
RCT: Randomized controlled trial
SAE: Serious adverse event
SD: Standard deviation
